# Determinants of parental vaccine hesitancy in Canada: results from the 2017 Childhood National Immunization Coverage Survey

**DOI:** 10.1186/s12889-023-17079-4

**Published:** 2023-11-24

**Authors:** Ruoke Chen, Mireille Guay, Nicolas L. Gilbert, Eve Dubé, Holly O. Witteman, Hina Hakim

**Affiliations:** 1 Infectious Diseases and Vaccination Programs Branch, Public Health Agency of Canada, Ottawa, ON Canada; 2 University of Montreal School of Public Health, Quebec, Canada; 3 Institut National de Santé Publique du Québec, Quebec, Canada; 4 Department of Anthropology, Laval University, Quebec, Canada; 5 Faculty of Medicine, Laval University, Quebec, Canada

**Keywords:** Parental vaccine hesitancy, Parental vaccine refusal, Childhood immunization

## Abstract

**Background:**

In 2019, the World Health Organization (WHO) designated vaccine hesitancy as one of the ten leading threats to global health. Vaccine hesitancy exists when vaccination services are available and accessible, but vaccine uptake is lower than anticipated. It is often attributed to lack of trust in vaccine safety and effectiveness, or low level of concern about the risk of many vaccine-preventable diseases. This study aimed to examine the sociodemographic factors associated with parental vaccine hesitancy and vaccine refusal in Canada using data from the 2017 Childhood National Immunization Coverage Survey (CNICS).

**Method:**

The 2017 CNICS was a cross-sectional and nationally representative survey to estimate national vaccine uptake and to collect information about parents’ Knowledge, Attitudes and Beliefs (KAB) regarding vaccination. Using the KAB questions, parental vaccine hesitancy (i.e., parental hesitation, delay or refusal of at least one recommended vaccination) and refusal (i.e., unvaccinated children) by sociodemographic factors was estimated using weighted prevalence proportions. A multinomial logistic regression model was fitted to examine associations between parental vaccine hesitancy or refusal and sociodemographic factors among parents of two-year-old children in Canada. Adjusted odds ratios (aOR) of being vaccine-hesitant or vaccine-refusing versus being non-vaccine-hesitant were generated.

**Results:**

Both unadjusted and adjusted logistic regressions models showed that parents with lower household income (aOR 1.7, 95% CI 1.2–2.5), and those with a higher number of children in the household (aOR 2.2, 95% CI 1.4–3.5) had higher vaccine hesitancy. Conversely, lower vaccine hesitancy was observed among immigrant parents (aOR 0.4, 95% CI 0.3–0.6). In addition, lower household income (aOR 4.0, 95% CI 1.3–12.9), and higher number of children in the household (aOR 6.9, 95% CI 2.1–22.9) were significantly associated with parental vaccine refusal. Regional variations were also observed.

**Conclusion:**

Several sociodemographic determinants are associated with parental vaccine hesitancy and refusal. The findings of the study could help public health officials and policymakers to develop and implement targeted interventions to improve childhood vaccination programs.

## Introduction

Despite vaccination being recognized as the most effective tool available for preventing infectious diseases and their complications [1, 2], there has been a growing number of individuals perceiving that vaccination is unsafe and unnecessary [3]. This intensified level of concern often results in an increased number of people delaying or refusing vaccines [3, 4]. In 2019, the World Health Organization (WHO) has designated vaccine hesitancy as one of the ten leading threats to global health [5]. In many countries, including Canada, parents hesitating to vaccinate their children has contributed to suboptimal rates of childhood vaccination, with associated sporadic outbreaks of vaccine-preventable diseases, including measles [6, 7] and pertussis [8–10].

Vaccine hesitancy defined by the World Health Organization as “a delay in acceptance or refusal of safe vaccines despite availability of vaccine services”, is recognized as a continuum between active demand for vaccine (pro-vaccine) and complete vaccine refusal (anti-vaccine) [11]. A range of factors could contribute to vaccine hesitancy such as doubts about the safety and effectiveness of approved and available vaccines, low levels of concern about the risk of many vaccine-preventable diseases, and the convenience of vaccination services, including the accessibility of information on vaccines [11, 12].

In Canada, young children are routinely vaccinated against diphtheria, pertussis (whooping cough), tetanus, poliomyelitis, *Haemophilus influenzae* type B (Hib), measles, mumps, rubella, varicella (chickenpox), meningococcal and pneumococcal infections, hepatitis B, influenza, and rotavirus [13]. To ensure widespread protection, a high vaccination coverage goal of 95% as part of the National Immunization Strategy objectives for 2016–2021 has been established for all childhood vaccines by two and seven years of age [14]. However, according to the Childhood National Immunization Coverage Surveys (CNICS), vaccine uptake remains suboptimal in Canada [15–17]. The CNICS is a national representative survey conducted consistently by the Public Health Agency of Canada (PHAC) and Statistics Canada to collect information on national immunization coverage for vaccines administered to children. Based on the 2017 CNICS, an estimated 2.4% of two-year-old children in Canada had not received any vaccine. The main reason for this, mentioned by 54% of non-vaccinating parents, was concern about vaccine safety and 33% were not confident in the usefulness or the effectiveness of vaccines [16]. Compared with vaccine refusers, vaccine-hesitant parents are a larger group whose children are under-vaccinated or have received all recommended doses but with delays. The prevalence of vaccine hesitancy among parents in Canada estimated using the information gathered from questions on knowledge, attitudes and beliefs (KAB) towards vaccines in the 2017 CNICS as 17% [18].

To better understand parental vaccine hesitancy and vaccine refusal in the Canadian context, this study aimed to (1) assess and compare prevalence of vaccine hesitancy and vaccine refusal by sociodemographic factors among parents of two-year-old children in Canada and to (2) examine the sociodemographic factors associated with vaccine hesitancy and vaccine refusal for routine childhood vaccines using the 2017 CNICS data. The 2017 CNICS data were used for the present analysis because the KAB sections of the questionnaire were re-designed in collaboration with a panel of experts to more accurately measure parental vaccine hesitancy and identify vaccine-hesitant parents. Furthermore, the dataset's larger sample size within the two-year-old age group facilitated an in-depth exploration of parental vaccine hesitancy and refusal across various sociodemographic factors, ensuring the national representativeness of the study's findings. Importantly, this comprehensive dataset will serve as a valuable comparative reference point to assess any changes in attitudes toward vaccination that may have arisen as a consequence of the COVID-19 pandemic. Quantifying the level of parental vaccine hesitancy nationally and identifying the factors underlying vaccine hesitancy and refusal are critical in guiding the effectiveness of future interventions to counter under-vaccination.

## Data and methods

### Study design

The 2017 CNICS was a cross-sectional, voluntary survey conducted by the Public Health Agency of Canada (PHAC) and Statistics Canada primarily to estimate national vaccine uptake for all publicly funded routine childhood vaccinations in Canada. The survey also assessed knowledge, attitudes, and beliefs among parents to better understand factors influencing decisions on vaccination for their children. Results pertaining to coverage estimates are described in a separate report [17]. A detailed description of sampling, data collection and data processing methods used in CNICS is available on the Statistics Canada’s website [19].

### Sample selection

The sampling frame of the survey was built using the list of children for whom the Canadian Child Benefit (CCB) was claimed as of June 2017. This list is estimated to include 96% of Children in Canada. Children aged two, seven, fourteen or seventeen years as of March 1, 2017, were eligible for inclusion in the survey. Children were randomly selected from the sampling frame by Statistics Canada. The sampling method ensured that only one eligible child from each household was selected. Children were selected by strata defined by age, provinces and territories. Sampling weights were assigned to respondents in order for estimates to be nationally representative of the Canadian population as of March 2017. The weights were adjusted to reduce non-response bias.

The target population for the present analysis consisted of parents of children in Canada aged two years only, except for institutionalized children and First Nations children living on reserve. The reason to restrict the analysis to one age group only was to ensure a homogeneous population with respect to the vaccines and number of doses recommended and offered to their children. In addition, the two-year-old age group had by far the largest sample size of CNICS, which permitted sufficient sample for a more comprehensive analysis on determinants of parental vaccine hesitancy and refusal.

While our sample is selected and weighted to reflect two-year-old children in Canada, our analysis primarily focuses on the parents of these children and their attitudes towards childhood vaccination. Therefore, when we refer to 'respondents' or 'parents' in our results, we are discussing the characteristics and behaviors of parents within the sampled households, as the data collection primarily involved parents' responses regarding vaccination decisions for their children.

### Data collection and processing

Survey data were collected through a computer assisted telephone interview (CATI) between November 22, 2017, and February 24, 2018. The person most knowledgeable about the child’s vaccinations, usually a parent or guardian (hereafter referred to as respondent/parent) was selected to answer the survey. This was followed by an assessment of the child’s healthcare record from the child’s immunization provider when it was available [16]. The entire questionnaire was reviewed and tested by Statistics Canada’s Questionnaire Design Resource Centre.

The questionnaire included several blocks of questions collecting information about parents’ KAB regarding vaccination. The KAB questions used to estimate vaccine hesitancy were asked only to respondents who provided a positive answer to the main question “Has your child ever been vaccinated?”, to which the respondents were not allowed to refuse to answer or respond “I don’t know”. Those parents who never vaccinated their child were considered as vaccine refusing and skipped the KAB questions on vaccine hesitancy. Those who had vaccinated their child at least once were asked “Have you ever decided not to immunize your child with a particular vaccine?” with the possibility to answer “Yes”, “No”, and “I don’t know”. Those who answered “Yes” were then asked which vaccine(s) they decided not to give to the child. Parents could answer this question by naming either antigens (e.g., measles, pertussis) or vaccines (e.g., MMR, DTaP). In the next block, respondents were asked “Have you ever been reluctant or hesitated to get a vaccination for your child?” with the possibility to answer “Yes”, “No”, and “I don’t know”. Respondents who answered “Yes” to this question were asked to identify the vaccine(s) they were reluctant to get for their child, the reasons why they hesitated and finally, what made them decide to have their child vaccinated despite their initial reluctance. Then, parents were also asked “Have you ever decided to delay any vaccines for your child?” with the possibility to answer “Yes”, “No” and “I don’t know”. Those who answered “Yes” were asked to choose the reasons why they decided to delay some vaccines for their child. The full survey questionnaire is available elsewhere [19].

### Quantitative variables

The CNICS is primarily aimed at measuring vaccination coverage. Therefore, any person able to provide information on the vaccination of the selected child can be accepted as a respondent. However, for the analysis of KAB, it is important to focus on the respondents who are most likely to be the ones making decisions regarding the child’s vaccination. For this reason, the analysis included only respondents who identified themselves as a biological parent, an adoptive parent or a step-parent to the selected child.

The total response rate of the 2017 CNICS for two-year-old children was 62%. Of the 6,502 two-year-old surveyed children, 6,463 (99%) had a biological parent, adoptive parent or step-parent as their respondent, whereas 39 (1%) had another person (e.g., older sibling, grandparent, or foster parent). Only the former were included in the analysis.

#### Outcome variables

The three-level nominal outcome variable on parental vaccine hesitancy was categorized as “Non-vaccine-hesitant”, “Vaccine-hesitant” and “Vaccine-refusing” parents. Parents whose child had not received any vaccine were classified as “vaccine refusers”. Those parents who have ever refused or been reluctant to get their child vaccinated for a particular vaccine other than influenza vaccine, or ever decided to delay any vaccines for a reason other than child having health issues were considered as “vaccine hesitant”. The refusal of influenza vaccine only, or reluctance about this vaccine only was not included in the definition of vaccine hesitancy because parents could perceive it differently from other childhood vaccines since each year the flu vaccine needs to be given again. Its content and effectiveness vary, and it addresses a disease that is often perceived as minor compared with other childhood diseases [20]. Moreover, not all provinces and territories included influenza vaccine in their routine childhood immunization schedule, each province and territory has designed and adopted its own approach to immunizing their population regarding this vaccine [21, 22]. Parents who never refused or hesitated to get their child vaccinated, but delayed vaccination due to child’s health issues only, were not considered as vaccine hesitant because this constitutes postponing a vaccination appointment for a sick child until he or she gets better.

#### Independent variables

Sociodemographic factors comprising parent’s gender, annual household income, level of education, region of residence, marital status, immigration status, child’s Indigenous status, number of children living in the household and parent’s age at child’s birth were included as independent variables. Variables included in the models have been previously demonstrated to be related to the modelled outcome or are considered to conceptually have a potential association with the outcome. For variables that had categories with largely unequal sample sizes (e.g. gender of PMK, marital status, immigration status and child’s Indigenous status), the largest category was used as a reference to mitigate potential issues arising from using a smaller group as the reference, such as unstable estimates and reduced statistical reliability. In cases where the sample sizes among categories were more balanced, or the variables exhibit a sequential or ordinal order in response options, the category that is either at the beginning or the end of the spectrum (e.g., education, income, number of children living in the household and parent’s age at child’s birth) or the category with the lowest odds to provide odds ratios (ORs) greater than 1 (e.g. provinces and territories) was selected for easier interpretation (See Table 1 for the definition of the categories for each variable.)

### Statistical analysis

Unweighted frequencies and proportions were computed to provide a description of the full sample. Parental vaccine hesitancy and refusal by sociodemographic factors was estimated using weighted prevalence proportions. A multinomial logistic regression model was fitted to examine the sociodemographic determinants associated with parental vaccine hesitancy and vaccine refusal. Adjusted odds ratios (aOR) of being vaccine-hesitant or vaccine-refusing versus being non-vaccine-hesitant were generated. The respondents who refused to answer or answered “I don’t know” to a given question of the survey were regrouped as “unknown” category and excluded from the prevalence calculations and the multinomial logistic model. The final sample size for the model was 6,243 with less than 3.5% of the sample being removed from the analysis, largely due to the unknown category in the outcome and independent variables. However, since there is a high proportion of unknowns for the education variable (16%), they were not excluded in the logistic regression models as opposed to the unknown category for other independent variables. Since no useful information can be drawn from this unknown education level category, it was left out of results tables beyond the one describing the sample. Simple logistic regression models were also performed to obtain unadjusted associations and compute unadjusted odds ratios for comparison with the adjusted values. Because the number of observations in some categories was too small to allow reliable estimates, marital status and child's Indigenous status variables were not included in the multiple regression models.

Variance and weighted 95% confidence intervals were estimated using the bootstrap method to account for the complex sampling design [23]. The precision of the estimates was determined by the coefficient of variation (CV). Proportion estimates with a CV from 16.6% to 33.3% indicated higher sampling error and should be interpreted with caution (E). Estimates with a coefficient of variation greater than 33.3% were considered unreliable and therefore were not reported (F). Analysis was completed using SAS EG 8.3 and the SURVEYFREQ and SURVEYLOGISTIC procedures were exclusively used to account for the complex survey design.

#### Ethics approval

The survey was carried out in compliance with the Statistics Act and other applicable laws and regulations. All experimental protocols were approved by Statistics Canada’s Office of Privacy Management and Information Coordination and its Data Ethics Secretariat, which apply many of the same criteria as an IRB when reviewing requests for datasets. In addition, the Health Canada and Public Health Agency of Canada Research Ethics Board was consulted as they would be the IRB of record for this study. This study is exempt from REB review pursuant to Article 2.2 of the Tri-Council Policy Statement on Ethical Conduct for Research Involving Humans [24].

## Results

### Demographics

The study sample consisted of 6,463 parents of children aged two years old living in Canada. Most respondents were female (91.2%). More than a third (43.1%) had a household income of $100,000 or greater and almost half (46.2%) were university graduates, indicating highly educated respondents. Only 13.5% were single parents, and 10.5% of children were identified as Indigenous. Most of the respondents were non-immigrants (77.8%). In addition, more than half of the respondents (51.9%) were living with 2 children aged 10 years or less in the same household. More parents (35.3%) were between 30 to 34 years when their child was born (Table 1).

**Table 1:** Characteristics of survey respondents

**Sociodemographic factors**	**Unweighted**		**Weighted**
	**Frequency (n)**	**Percent (%)**	**Percent (%)**
**Parental vaccine hesitancy**			
Non-hesitant parents	5214	81.0	80.2
Vaccine-hesitant parents	1092	17.0	17.4
Vaccine-refusing parents	122	2.1	2.4
Unknown	25	0.0	-
**Gender of PMK**^**a**^			
Male	567	8.8	11.6
Female	5896	91.2	88.4
**Household income**			
Less than 50,000$	1599	24.7	28.9
Between 50,000$ and 100,000$	2083	32.2	32.5
Between 100,000$ and 150,000$	1452	22.5	21.4
Above 150,000$	1329	20.6	17.3
**Highest level of education in the household**			
Secondary or less	895	13.9	12.6
Post secondary	1557	24.1	24.1
University Graduate	2983	46.2	47.3
Unknown	1028	15.9	16.0
**Provinces and territories**			
Atlantic	2149	33.3	5.4
Quebec	629	9.7	22.5
Ontario	641	9.9	38.0
Manitoba	556	8.6	4.0
Saskatchewan	568	8.8	3.6
Alberta	624	9.7	14.4
British Columbia	579	9.0	11.5
Territories	717	11.1	0.5
**Marital Status**			
Single parent	874	13.5	13.3
Non-single parent/Unknown^b^	5589	86.5	86.7
**Immigration status**			
Immigrant parent	1350	20.9	33.6
Non-immigrant parent	5029	77.8	66.4
Unknown	84	1.3	-
**Child's Indigenous status**			
Indigenous	680	10.5	4.7
Non-Indigenous	5660	87.6	95.3
Unknown	123	1.9	-
**Number of children in the household**			
**Aged 10 years or less**			
1 Child	1622	25.1	24.2
2 Children	3354	51.9	52.2
3 Children	1041	16.1	17.6
4 Children or more	358	5.5	6.0
Unknown	88	1.4	-
**Parent's age when child was born**			
25 years and under	1111	17.2	22.8
26 to 29 years old	1558	24.1	30.1
30 to 34 years old	2283	35.3	47.1
35 years and older	1368	21.2	23.5
Unknown	143	2.2	-

^a^*PMK* Person Most Knowledgeable of the child's vaccination (birth, step or adoptive parents)

^b^Unknown category is aggregated with Non-single parent in Table 1 due to confidentiality reasons (counts < 15)

### Prevalence estimates

#### Parental vaccine hesitancy

Overall, the proportion of vaccine-hesitant parents was 17.4% (Table 1). When looking at the population distribution by sociodemographic factors in Table 2, non-immigrant parents (20.9%), those having 4 young children or more (28.5%) had significantly higher proportions of vaccine hesitancy. Regional variation in parental vaccine hesitancy prevalence was also observed. Comparing to Atlantic region, the proportion of vaccine-hesitant parents was higher in Quebec (23.9%), and in the Territories (21.9%). (Table 2).

**Table 2:** Parental vaccine hesitancy status by sociodemographic factors

**Sociodemographic factors**	**Non-vaccine-hesitant**	**Vaccine-hesitant**	**Vaccine-refusing**
	**%(95%CI)**	**%(95%CI)**	**%(95%CI)**
**Gender of PMK**^**a**^			
Male	86.8(82.9–90.7)	9.3(5.7–12.9)E	3.8(1.9–5.7)E
Female	79.4(77.7–81.0)	18.5(16.9–20.0)	2.2(1.6–2.8)
**Household income**			
Less than 50,000$	77.4(74.2–80.7)	19.2(16.2–22.3)	3.3(2.0–4.6)E
Between 50,000$ and 100,000$	80.4(77.8–83.0)	17.2(14.7–19.7)	2.3(1.3–3.3)E
Between 100,000$ and 150,000$	80.3(76.9–83.6)	17.4(14.2–20.7)	2.3(1.1–3.5)E
Above 150,000$	84.4(81.3–87.4)	14.7(11.6–17.7)	1.0(0.4–1.6)E
**Highest level of education in the household**			
Secondary or less	78.2(73.6–82.7)	17.6(13.5–21.8)	4.2(2.2–6.2)E
Post secondary	78.7(75.5–81.9)	18.9(15.7–22.0)	2.4(1.3–3.5)E
University Graduate	82.4(80.2–84.5)	15.7(13.5–17.8)	1.9(1.1–2.8)E
**Provinces and territories**			
Atlantic	83.0(81.2–84.8)	15.6(13.9–17.4)	1.4(0.8–1.9)E
Quebec	73.1(69.5–76.7)	23.9(20.5–27.3)	3.0(1.6–4.3)E
Ontario	82.7(79.7–85.6)	15.6(12.8–18.5)	1.7(0.7–2.7)E
Manitoba	81.0(77.7–84.3)	15.7(12.6–18.7)	3.4(1.9–4.8)E
Saskatchewan	82.0(77.8–86.2)	15.3(11.8–18.7)	2.8(1.3–4.2)E
Alberta	83.6(80.7–86.6)	14.3(11.6–17.1)	2.0(0.9–3.2)E
British Columbia	79.9(76.6–83.3)	16.2(12.9–19.4)	3.9(2.3–5.6)E
Territories	76.3(72.8–79.8)	21.9(18.4–25.3)	1.8(0.7–2.9)E
**Marital status**			
Single parent	76.3(71.3–81.2)	F	F
Non-single parent	80.9(79.2–82.5)	16.8(15.2–18.4)	2.4(1.7–3.1)
**Immigration status**			
Immigrant parent	87.2(84.7–89.6)	10.7(8.4–13.0)	2.1(1.1–3.1)E
Non-immigrant parent	76.5(74.5–78.5)	20.9(19.1–22.8)	2.6(1.8–3.3)
**Child’s Indigenous status**			
Indigenous	75.4(68.2–82.7)	F	F
Non-Indigenous	80.3(78.7–81.9)	17.3(15.8–18.9)	2.3(1.7–3.0)
**Number of children in the household**			
**Aged 10 years or less**			
1 Child	83.2(80.3–86.2)	F	F
2 Children	80.3(78.1–82.4)	18.0(15.9–20.1)	1.7(1.1–2.4)E
3 Children	79.7(76.2–83.2)	15.1(11.9–18.3)	5.2(3.0–7.4)E
4 Children or more	66.1(58.2–74.0)	28.5(20.8–36.2)	5.4(2.4–8.4)E
**Parent’s age when child was born**			
25 years and under	77.6(73.2–82.0)	19.6(15.3–23.8)	2.8(1.1–4.5)E
26 to 29 years old	79.4(76.2–82.5)	18.2(15.2–21.2)	2.4(1.3–3.5)E
30 to 34 years old	80.0(77.5–82.5)	17.9(15.4–20.3)	2.1(1.1–3.1)E
35 years and older	82.7(79.6–85.9)	14.7(11.7–17.8)	2.5(1.3–3.7)E

^a^*PMK* Person Most Knowledgeable of the child’s vaccination (birth, step or adoptive parents)

E Estimate is of marginal quality, use with caution

F Estimate is suppressed due to data quality concerns or confidentiality reasons

Note: Unknown categories were excluded from the prevalence estimates

#### Parental vaccine refusal

In total, 2.4% of children had never been vaccinated (Table 1). Parents with household income less than $50,000 had a significantly higher rate of vaccine refusal (3.3%) comparing to those with household income above $150,000 (1.0%) (Table 2). Compared to parents having only 1 child in the household, those having 3 or 4 young children had significantly higher rates of vaccine refusal (5.2% and 5.4%, respectively). Moreover, the proportion of vaccine-refusing parents was higher in British Columbia (3.9%) than in Atlantic region (1.4%) (Table 2).

### Determinants of parental vaccine hesitancy and refusal

#### Parental vaccine hesitancy

Both unadjusted and adjusted logistic regressions models showed that parent’s gender, household income, region of residence, immigration status, and number of children in the household were significantly associated with vaccine hesitancy (Table 3). Specifically, being a male responding parent, as well as being an immigrant parent were associated with lower odds of being vaccine-hesitant (aOR 0.6, 95% CI 0.4–0.9 and aOR 0.4, 95% CI 0.3–0.6, respectively). Higher parental vaccine hesitancy was observed in parents with household income less than $50,000 (aOR 1.7, 95% CI 1.2–2.5) and in parents having 4 young children or more (aOR 2.2, 95% CI 1.4–3.5). Compared to parents in the Atlantic region, the odds of being vaccine hesitant were greater for those living in Quebec, Ontario, British Columbia and in the Territories (Table 3).

**Table 3:** Associations between sociodemographic factors and parental vaccine hesitancy status

	**Outcome 'Vaccine-hesitant'**						**Outcome 'Vaccine-refusing'**	
	**Reference: Non-vaccine-hesitant**							
	**Simple logistic regression**		**Multiple logistic regression**		**Simple logistic regression**		**Multiple logistic regression**	
**Sociodemographic factors**	**OR (95%CI)**	***p*-value**	**aOR (95%CI)**	***p*-value**	**OR (95%CI)**	***p*-value**	**aOR (95%CI)**	***p*-value**
**Gender of PMK**^**a**^								
Male	**0.5(0.3–0.7)**	** < .001**	**0.6(0.4–0.9)**	**0.011**	1.6(0.9–3.0)	0.130	1.7(0.8–3.5)	0.154
Female	Reference		Reference		Reference		**Reference**	
**Household income**								
Less than 50,000$	**1.4(1.0–1.9)**	**0.024**	**1.7(1.2–2.5)**	**0.006**	**3.8(1.5–9.3)**	**0.004**	**4.0(1.3–12.9)**	**0.019**
Between 50,000$ and 100,000$	1.2(0.9–1.6)	0.157	1.3(1.0–1.8)	0.091	**2.5(1.1–6.1)**	**0.037**	2.6(1.0–7.2)	0.058
Between 100,000$ and 150,000$	1.3(0.9–1.7)	0.177	1.2(0.8–1.7)	0.302	2.5(1.0–6.4)	0.054	2.5(1.0–6.8)	0.062
Above 150,000$	Reference		Reference		Reference		Reference	
**Highest level of education in the household**								
Secondary or less	1.2(0.8–1.7)	0.325	1.0(0.7–1.4)	0.887	**2.3(1.1–4.7)**	**0.022**	1.4(0.6–3.3)	0.452
Post secondary	1.3(1.0–1.6)	0.076	1.0(0.8–1.4)	0.824	1.3(0.7–2.5)	0.413	1.1(0.5–2.2)	0.823
University Graduate	Reference		Reference		Reference		Reference	
**Provinces and territories**								
Atlantic	Reference		Reference		Reference		Reference	
Quebec	**1.7(1.4–2.2)**	** < .001**	**2.1(1.6–2.6)**	** < .001**	**2.5(1.2–5.1)**	**0.011**	**2.5(1.2–5.3)**	**0.015**
Ontario	1.0(0.8–1.3)	0.970	**1.3(1.0–1.7)**	**0.029**	1.2(0.5–2.9)	0.612	1.3(0.5–3.3)	0.529
Manitoba	1.0(0.8–1.3)	0.844	1.2(0.9–1.5)	0.328	**2.5(1.3–5.0)**	**0.006**	**2.1(1.1–4.2)**	**0.034**
Saskatchewan	1.0(0.7–1.4)	0.945	1.1(0.8–1.6)	0.525	2.1(0.9–4.8)	0.091	1.5(0.6–3.9)	0.395
Alberta	0.9(0.7–1.2)	0.464	1.1(0.9–1.5)	0.358	1.5(0.7–3.4)	0.337	1.7(0.7–3.8)	0.228
British Columbia	1.1(0.8–1.4)	0.612	**1.5(1.1–1.9)**	**0.008**	**3.0(1.5–6.0)**	**0.002**	**3.9(1.9–7.8)**	**0.000**
Territories	**1.5(1.2–1.9)**	**0.001**	**1.6(1.2–2.1)**	**0.003**	1.4(0.7–3.2)	0.364	0.9(0.4–2.3)	0.875
**Marital status**^**b**^								
Single parent	1.4(1.0–1.9)	0.055			1.0(0.4–2.4)	0.975		
Non-single parent	Reference				Reference			
**Immigration status**								
Immigrant parent	**0.4(0.3–0.6)**	** < .001**	**0.4(0.3–0.6)**	** < .001**	0.7(0.4–1.4)	0.325	0.6(0.3–1.2)	0.143
Non-immigrant parent	Reference		Reference		Reference		Reference	
**Child's Indigenous status**^**b**^								
Indigenous	1.3(0.9–2.1)	0.196			1.2(0.4–4.0)	0.734		
Non-Indigenous	Reference				Reference			
**Number of children in the household**								
**Aged 10 years or less**								
1 Child	Reference		Reference		Reference		Reference	
2 Children	1.2(0.9–1.5)	0.195	1.2(0.9–1.5)	0.233	1.7(0.7–4.1)	0.279	1.9(0.7–5.1)	0.206
3 Children	1.0(0.7–1.4)	0.972	1.0(0.7–1.5)	0.804	**4.9(1.9–13.1)**	**0.001**	**5.6(1.9–16.0)**	**0.002**
4 Children or more	**2.3(1.5–3.6)**	** < .001**	**2.2(1.4–3.5)**	**0.001**	**6.2(2.2–17.5)**	**0.001**	**6.9(2.1–22.9)**	**0.002**
**Parent's age when child was born**								
25 years and under	1.4(1.0–2.0)	0.055	1.0(0.7–1.5)	0.908	1.2(0.5–2.7)	0.662	1.0(0.4–2.5)	0.928
26 to 29 years old	1.3(0.9–1.8)	0.117	1.0(0.7–1.4)	0.991	1.0(0.5–2.1)	0.998	0.9(0.4–1.9)	0.702
30 to 34 years old	1.3(0.9–1.7)	0.124	1.0(0.8–1.4)	0.771	0.9(0.4–1.8)	0.710	0.8(0.4–1.8)	0.613
35 years and older	Reference		Reference		Reference		Reference	

^a^*PMK* Person Most Knowledgeable of the child's vaccination (birth, step or adoptive parents)

^b^ Variable excluded from the multiple logistic regression due to small number of observations

Bold values indicate significant odds ratios after adjustment at the 5% level

Note: Unknown categories were excluded from the logistic regression models for all variables except Education due to high proportion of Unknown category (16%). Since no useful information can be drawn from this unknown education level category, it was left out of the result table

#### Parental vaccine refusal

In simple logistic regression, household income, level of education, region of residence, and the number of children in the household were significantly associated with parental vaccine refusal. In the adjusted logistic regression model, only household income, region of residence, and the number of children in the household appeared as significant determinants. The odds of being a vaccine-refusing parent were significantly higher among those with a household income of less than $50,000 compared to those with a household income above $150,000 (aOR 4.0, 95% CI 1.3–12.9). Similarly, having 3 or 4 children or more in a household was significantly associated with higher odds of vaccine refusal (aOR 5.6, 95% CI 1.9–16.0 and aOR 6.9, 95% CI 2.1–22.9, respectively). Compared to parents in the Atlantic region, the odds of being vaccine refusing was higher among those living in Quebec, Manitoba, and British Columbia (Table 3).

## Discussion

### Parental vaccine hesitancy

Overall, the estimated prevalence of parental vaccine hesitancy in Canada among children aged two years old was 17.4%. In this analysis, factors associated with childhood vaccine hesitancy were parent’s gender, household income, region of residence, immigration status, and number of children in the household. Compared to female responding parents, male responding parents had lower odds of vaccine hesitancy, which was consistent with findings from previous literature that mothers were more likely to be vaccine hesitant than fathers [25, 26]. This could be related to women being more likely to express concern about vaccine safety than men [27]. In this study, the association between parent's gender and vaccine hesitancy should be interpreted with caution since the decision to vaccinate a child or not is often a joint decision made by both parents. In addition, vaccine hesitancy was lower among immigrant parents. This was consistent with a similar Canadian study showing that recent immigration to Canada decreased likelihood of refusal, delay, or reluctance [28]. However, another study showed that vaccine hesitancy was more common among non-Canadian-born parents, who cited concerns about vaccine safety, efficacy, and necessity as reasons for not vaccinating their children [29].

Higher vaccine hesitancy was observed in parents with lower household income and in parents living with 4 or more young children in the same household. A similar national study in US demonstrated that lower household income predicted hesitancy about routine childhood and influenza vaccines [20]. A few other national studies have also found lower income to be associated with higher levels of concern about the safety or necessity of vaccines [27, 30, 31]. In addition, a few studies found that delaying vaccination has been associated with having a larger number of children in the household [32, 33]. This could be associated with concerns about the number and timing of vaccinations required. Also, it may due to larger families being associated with higher low-income rate as women in lower income groups tend to have higher birth rates [34]. These factors may also combine to decrease vaccine uptake for logistical reasons. Specifically, families with multiple children and limited disposable income may have more difficulty in getting all children to all recommended appointments.

### Parental vaccine refusal

In this analysis, household income, region of residence, and the number of children in the household appeared as significant determinants of non-vaccination. The odds of being a vaccine-refusing parent were higher among those with lower household income. This was consistent with findings in the previous publications that low income is one of the factors associated with under-vaccination among children [35, 36]. One reason for this may be that families with lower incomes could face more barriers to accessing public health services, including vaccination [27] Although, all recommended vaccinations are free of charge in Canada, taking children to appointments may incur costs due to transportation, and time off work, both for the appointment itself and in the event that the child experiences a fever following the vaccine.

Having 3 or 4 children or more in a household was significantly associated with higher odds of being vaccine refusing. Previous studies have shown the number of children in a family to be associated with vaccine refusal. Parents with more children are more likely to refuse vaccines due to concerns about the safety and efficacy of vaccines [37–39].

The regional differences in parental vaccine hesitancy and refusal observed in this study could be associated with the differences in immunization schedules and program delivery between provinces and territories. Since not all jurisdictions have designed and adopted the same approach to immunizing their population regarding childhood vaccines, the importance of certain vaccines may be perceived differently among parents from different regions.

### Strengths and limitations

The present study has several strengths and limitations to consider when interpreting the results. The major strength of the survey was the sufficiently large sample size to allow for analysis of parental vaccine hesitancy and refusal by several sociodemographic factors. Additionally, given the complex survey design and the use of survey weights, the findings are ensured to be nationally representative and allow us to make inferences to all children in Canada.

There are some limitations with the study that need to be acknowledged. First, self-reported data sources from CNICS are susceptible to bias, such as social desirability bias, recall bias, and non-response bias. To mitigate them, rigorous quality assurance mechanisms were applied by Statistics Canada during the interviews and across all steps of the statistical process. Moreover, the survey was designed primarily to measure vaccination coverage and the methodology used may not be ideal for measuring parental vaccine hesitancy. The sampling strategy was designed to yield a sample representative of two-year-old children living in Canada, not of parents and guardians of two-year-old children. There may therefore be confounding based on the parents’ choice of who answered. For example, the male respondents might be different from all males parents of two-year-old children. In addition, the targeted respondents were those most knowledgeable of the child’s vaccination information, who may or may not be the one making decisions about vaccination. To mitigate this, we included in the analysis only those who were most likely to make decisions regarding their child’s vaccination, which included biological parents, adoptive parents and step-parents. Moreover, since only one parent was interviewed, it is important to consider that the parents of a child may hold different views about vaccination. The vaccination or non-vaccination of a child may therefore result from a compromise between the parents, or from one parent acting against the will of the other. In such cases, assuming a child’s partial, refused, or delayed vaccination as a surrogate of parental hesitancy may be inaccurate. Like many other Statistics Canada surveys, the CNICS excluded First Nations on-reserve communities and institutionalized children, and interviews were conducted in English or French, excluding children with parents that are not fluent in either official language. Furthermore, children in the child welfare system were not included on the CCB frame since foster parents cannot claim the CCB for the children under their care. These populations may have differences in KAB regarding vaccination and access or utilization of healthcare services from that of other children in Canada.

## Conclusion

In this study, parental vaccine hesitancy was higher in those with lower household income, non-immigrant parents, and those living in a household with a higher number of children. Similarly, lower household income, and higher number of children in the household were significantly associated with parental vaccine refusal. Regional variations were also observed. These findings could help public health officials and policymakers to develop and implement targeted interventions to improve childhood vaccination programs. However, since COVID-19 was first identified as a pandemic by the World Health Organisation (WHO) in March 2020, there has been worldwide attention placed on the development of safe and effective vaccines. It would be therefore important to consider the changes in attitude to vaccination as a result of the COVID-19 pandemic. Our future research will use the CNICS 2021 post-pandemic results to further investigate the impact of the COVID-19 pandemic on childhood vaccination.

## Data Availability

The dataset for the analysis can be accessed publicly through data access requests to Statistics Canada Research Data Centres, https://www.statcan.gc.ca/en/microdata/data-centres.

## Abbreviations

aOR: Adjusted odds ratio
CATI: Computer-assisted telephone interviewing
CCB: Canadian Child Benefit
CNICS: Childhood National Immunization Coverage Survey
CI: Confidence interval
COVID-19: Coronavirus disease of 2019
CV: Coefficient of variation
KAB: Knowledge, attitudes, and beliefs
OR: Odds ratio
PHAC: Public Health Agency of Canada
SAGE: Strategic Advisory Group of Experts
SAS: Statistical Analysis System
US: United States
WHO: World Health Organization
