# Correction: Determinants of parental vaccine hesitancy in Canada: results from the 2017 Childhood National Immunization Coverage Survey

**DOI:** 10.1186/s12889-024-20862-6

**Published:** 2025-03-27

**Authors:** Ruoke Chen, Mireille Guay, Nicolas L. Gilbert, Eve Dubé, Holly O. Witteman, Hina Hakim

**Affiliations:** 1 Infectious Diseases and Vaccination Programs Branch, Public Health Agency of Canada, Ottawa, ON Canada; 2 University of Montreal School of Public Health, Quebec, Canada; 3 Institut National de Santé Publique du Québec, Quebec, Canada; 4 Department of Anthropology, Laval University, Quebec, Canada; 5 Faculty of Medicine, Laval University, Quebec, Canada

**BMC Public Health (2023) 23:2327**

10.1186/s12889-023-17079-4

The original publication of this article contained a typo in the abstract. The incorrect and correct information is listed in this correction article. The original article has been updated.

Incorrect.

Both unadjusted and adjusted logistic regressions models showed that parents with lower household income (aOR 1.7, 95% CI 1.2–2.5), and those with a higher number of children in the household (aOR 2.2, 95% CI 1.4–3.5) had higher vaccine hesitancy. Conversely, lower vaccine hesitancy was observed among non-immigrant parents (aOR 0.4, 95% CI 0.3–0.6).

Correct.

Both unadjusted and adjusted logistic regressions models showed that parents with lower household income (aOR 1.7, 95% CI 1.2–2.5), and those with a higher number of children in the household (aOR 2.2, 95% CI 1.4–3.5) had higher vaccine hesitancy. Conversely, lower vaccine hesitancy was observed among immigrant parents (aOR 0.4, 95% CI 0.3–0.6).

